# Supplementary material for: Hybrid Models and Biological Model Reduction with PyDSTool
Source: PLoS Comput Biol. 2012 Aug 9;8(8):e1002628. doi: 10.1371/journal.pcbi.1002628 (PMC3415397; doi:10.1371/journal.pcbi.1002628)
Supplement: Text S4 — Complete source code for the PyDSTool package (version 0.88.120504). Includes API documentation and help files linking to web pages. This file is identical to the current public release on Sourceforge.net. (ZIP) [file pcbi.1002628.s004.zip › PyDSTool/html/PyDSTool.Toolbox.optimizers.helpers.finite_difference-module.html]

xml version="1.0" encoding="ascii"?


PyDSTool.Toolbox.optimizers.helpers.finite\_difference


| Home | Trees | Indices | Help | PyDSTool |
| --- | --- | --- | --- | --- |

Package PyDSTool ::
Package Toolbox ::
Package optimizers ::
Package helpers ::
Module finite\_difference

[hide private]

[frames] | no frames]

# Module finite\_difference

source code


|  |  |
| --- | --- |
| Classes | [hide private] |

FiniteDifferencesFunction

ForwardFiniteDifferences  
A function that will be able to computes its derivatives with a
forward difference formula

CenteredFiniteDifferences  
A function that will be able to computes its derivatives with a
centered difference formula

FiniteDifferencesCache  
General class for recognition by ParamEst as a function with a
non-explicit derivative.

ForwardFiniteDifferencesCache  
A function that will be able to computes its derivatives with a
forward difference formula.

| Home | Trees | Indices | Help | PyDSTool |
| --- | --- | --- | --- | --- |

|  |  |
| --- | --- |
| Generated by Epydoc 3.0.1 on Fri May 4 15:24:05 2012 | http://epydoc.sourceforge.net |
